# A systematic hybrid machine learning approach for stress prediction

**DOI:** 10.7717/peerj-cs.1154

**Published:** 2023-02-08

**Authors:** Cheng Ding, Yuhao Zhang, Ting Ding

**Affiliations:** 1 Emory University, Atlanta, GA, United States; 2 University of Nottingham, Nottingham, United Kingdom; 3 East China University of Technology, NAN Chang, China

**Keywords:** Machine learning, Stress detection, Hybrid appraoch

## Abstract

Stress is becoming an increasingly prevalent health issue, seriously affecting people and putting their health and lives at risk. Frustration, nervousness, and anxiety are the symptoms of stress and these symptoms are becoming common (40%) in younger people. It creates a negative impact on human lives and damages the performance of each individual. Early prediction of stress and the level of stress can help to reduce its impact and different serious health issues related to this mental state. For this, automated systems are required so they can accurately predict stress levels. This study proposed an approach that can detect stress accurately and efficiently using machine learning techniques. We proposed a hybrid model (HB) which is a combination of gradient boosting machine (GBM) and random forest (RF). These models are combined using soft voting criteria in which each model’s prediction probability will be used for the final prediction. The proposed model is significant with 100% accuracy in comparison with the state-of-the-art approaches. To show the significance of the proposed approach we have also done 10-fold cross-validation using the proposed model and the proposed HB model outperforms with 1.00 mean accuracy and +/−0.00 standard deviation. In the end, a statistical T-test we have done to show the significance of the proposed approach in comparison with other approaches.

## Introduction

Good mental health is important for a person’s overall well-being and their ability to achieve goals, however, there are many factors impacting mental health. According to a report round, about 190 million people face a higher level of stress (Kelly, 2021). This world was a sadder, more worried, angrier, and more stressful place in 2020 at any time in the last 15 years and a poll on stress concluded that 2020 “officially became the most stressful year in recent history” (Kelly, 2021). COVID-19 increased the rate of stress worldwide while other political issues are also involved in the stress level increase in public (Kelly, 2020). People in this fast-growing world are trying to perform better and they are not caring about their health issues. In this competitive world, there are also other factors affecting mental health recent COVID-19 outbreak, social media usage, business, work, education, *etc*., and cause numerous mental states such as stress, despair, anxiety, and depression. The most common of these is stress as according to a study approximately 30% people have mental health issues (Salari et al., 2020).

There are many manual methods are available to measure stress measure such as psychologists and psychiatrists physically examining the patients suffering from stress and correspondingly (Crosswell & Lockwood, 2020; Kim et al., 2018). They check their daily routine to measure the stress level of the patient (Crosswell & Lockwood, 2020). The examination can lead to some conclusions. There are disadvantages to this manual examination such as its costly and timely practice. The patient has to make an appointment with his psychologists and have to pay his fee. On fix date, the patient has to visit the psychologist for a routine checkup, and this process requires a lot of time by both patient and psychologist. This traditional method can be replaced with technology that can help to get better and cost-effective results in the meantime (Rustam et al., 2022).

Lots of studies from literature also used machine learning for stress detection such as the study by Jung & Yoon (2017), who worked on stress levels prediction as normal, low, middle, and high. They used random forest (RF), and support vector machine (SVM) as learning models trained on electroencephalography (EEG), electrocardiography (ECG), and respiration rate (RR) features. Similarly, Akmandor & Jha (2017) also worked on stress detection using machine learning models such as SVM and k nearest neighbor (KNN) for stress detection. This study also contributes to this regard and proposed an approach for stress detection using machine learning techniques. We used a hybrid machine learning model which consists of two machine learning models. To train the machine learning model we used some previous research data. Rachakonda et al. (2020, 2018) collected the data from literature and we used that for our experiments. Our proposed approach consists of the hybrid model which can generate more significant results for stress detection in terms of accuracy and efficiency.

- This study proposed an approach for stress detection which is more accurate and efficient in comparison state-of-the-art approaches.
- This study proposed a hybrid model that combined two machine learning models using soft voting criteria to achieve significant results in comparison to individual models on the used dataset.
- Our proposed approach with more several target classes for stress level prediction low/normal, medium-low, medium, medium-high, and high.
- This study contribute also in terms of efficiency which means the proposed approach gives more accurate results with low computational cost.
- Present extensive literature on stress detection and give a strong comparison between the state-of-the-art approaches.
- We have done a statistical T-test to show the significance of the proposed HB model.

The rest of the article is divided into four sections. “Related Work” contain the extensive literature review on stress detection and the dataset was used in the “Material and Methods”. “Results” presents the results of machine learning and deep learning models for stress detection and in the end, the conclusion is given in “Conclusion”.

## Related work

Stress detection is one of the most researchable areas related to mental health. Many researchers have done work in this domain and proposed several stress measure approaches but it is still an open area for other researchers to improve the prediction accuracy. Thelwall (2017) proposed a system known as TensiStrength to detect the level of stress and anxiety. They used a rule-based approach as a lexical technique to identify stress and anxiety. Lin et al. (2017) proposed a factor graph model combined with a CNN for stress detection. They used Twitter and SinaWeibo data to train learning models and achieved significant results. Have done machine learning approaches for stress detection such as the study by Schmidt et al. (2018), which deployed five machine learning models for stress detection as linear discriminant analysis (LDA), RF, decision tree, KNN and AdaBoost (ADA). They worked on several stress conditions such as baseline, amusement, meditation, *etc*.

Koldijk, Neerincx & Kraaij (2018) proposed an approach for stress detection using several human body features such as body postures and facial expressions. The deployed machine learning model SVM achieved 90% accuracy. Rizwan et al. (2019) also used SVM models for stress classification and used ECG features to achieve 98.6%. Ahuja & Banga (2019) proposed an approach for stress detection in university students using machine learning models. They used RF, LR, SVM, and other states of the art models while the SVM model performs significantly with the proposed approach to achieve significant 85.71% accuracy. Salazar-Ramirez et al. (2018) also proposed an approach for stress detection using galvanic skin response (GSR), heart rate (HR), and breath features. They worked on two target classes stressed and relax and used Gaussian SVM as machine learning models. GSR shows the highest rate for stress indication and SVM achieved an 80% accuracy score.

Alberdi et al. (2018), used heart rate, heart rate variability, psychological features, SCL, and behavioral features for stress detection. They worked on stressors, relaxed, pressure, and normal target classes and used SVM as a machine learning model. Lim et al. (2018), proposed a deep learning approach for stress detection using EEG data. The used dataset consists of two target classes stress and workload. The proposed model is significant in the used dataset. Mahajan (2018), proposed a feedforward neural network for stress detection using temporal and peak features of EEG data. They used two target class data consisting of normal and stress classes. The proposed model consists of 25 hidden layers and achieved significant 60% accuracy on the used dataset.

Bichindaritz et al. (2017) proposed an approach for stress detection using ECG, foot GSR, EMG, RR, and intermittent HR features. They also deployed several learning models including deep learning and machine learning. They used a dataset consisting of three target classes Low, medium, and high stress. They used MLP, RF, Naive Bayes, and k star models, and k star achieved a significant 100% accuracy score. The study Lee & Chung (2016), proposed an approach for stress detection using machine learning models. They used self-reports, standard deviation, median, SC mean, variance, and magnitude as the feature set. They deployed the SVM model on the stress and non-stress target classes dataset. SVM achieved a significant 94% accuracy score on the used dataset.

In this study, we proposed an ensemble model for stress detection. According to the literature, several studies in past also worked on ensemble learning approaches such as Khullar et al. (2022) proposed an ensemble model for stress detection using physiological signals based on anxiety. Issa (2021) used a two-step ensemble for stress detection in automobile drivers. Similarly, Di Martino & Delmastro (2020) also used an ensemble model for physiological stress prediction. Lee et al. (2022), proposed an ensemble model by combining deep learning models such as gated recurrent unit, CNN, and recurrent neural networks. They deployed the ensemble model for emotion detection using the tweets dataset.

Table 1 present the summary of the related work section and we concluded the literature with some findings. In previous studies, researchers mostly worked on two and three target classes but in this study, we consider five target classes for better stress level measurement. Second, we concluded in the literature that most of the studies used SVM models for classification which can perform better for large datasets while we proposed a hybrid model which is more efficient as compared to previous approaches.

**Table 1:** Related work summary.

Ref.	Approach	Model	Aim	Dataset/Features
Schmidt et al. (2018)	ML	LDA, RF, DT, AB	Stress detection using machine learning	Self-generated dataset using physical examination
Koldijk, Neerincx & Kraaij (2018)	ML	SVM	Stress detection using machine learning	Human body features such as body postures and facial expressions.
Rizwan et al. (2019)	ML	SVM	Stress detection using machine learning	ECG signals.
Ahuja & Banga (2019)	ML	SVM	Stress detection using machine learning	Self-generated dataset using physical examination
Salazar-Ramirez et al. (2018)	ML	Gaussian SVM	Stress detection using machine learning	GSR, HR, breath features
Alberdi et al. (2018)	ML	SVM	Stress detection using machine learning	Heart Rate, Heart Rate Variability, psychological features, SCL, and behavioral features
Lim et al. (2018)	DL	Artificial neural networks	Stress detection using machine learning	EEG data
Mahajan (2018)	DL	Feedforward neural network	Stress detection using machine learning	Temporal and peak features of EEG data
Bichindaritz et al. (2017)	ML	K star	Stress detection using machine learning	ECG, foot GSR, EMG, RR, and intermittent HR features
Lee & Chung (2016)	ML	SVM	Stress detection using machine learning	Self-reports, standard deviation, median, SC Mean, variance, magnitude as feature set

## Materials and Methods

**Figure 1:**
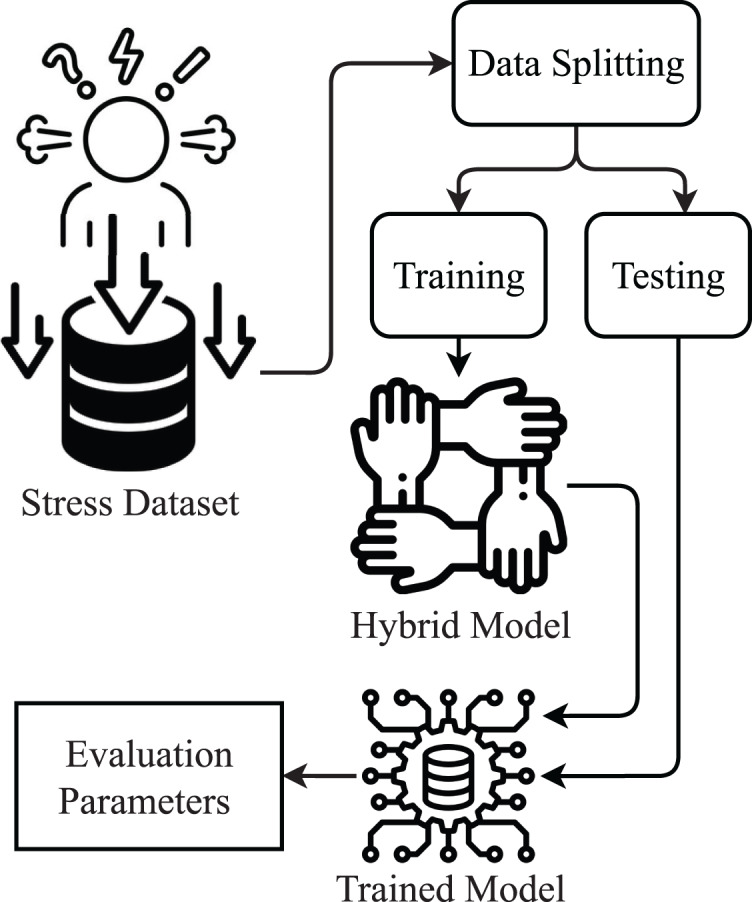
Proposed methodology diagram.

This study is about stress detection using a supervised machine learning approach. The proposed methodology diagram is shown in Fig. 1.

### Dataset description

**Figure 2:**
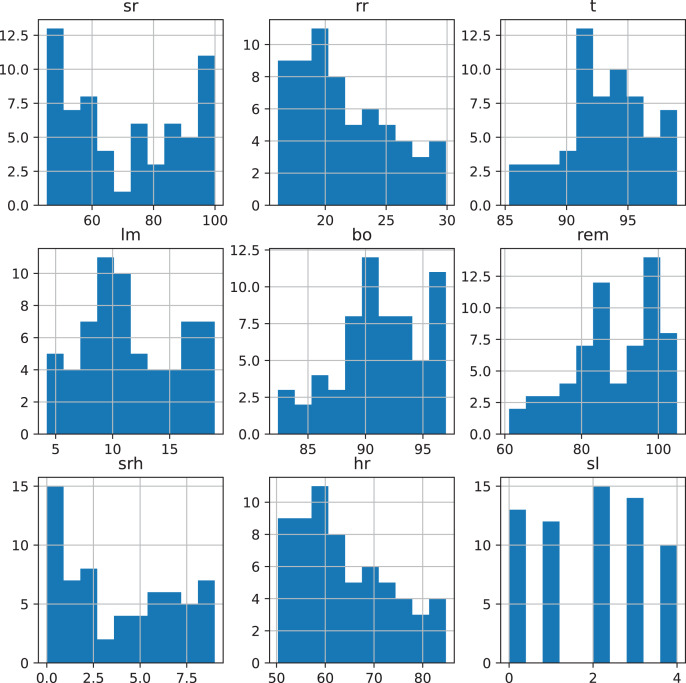
Dataset variables value ranges.

In our proposed approach first, we acquired the stress detection dataset from Kaggle (https://www.kaggle.com/code/ingridai/stress-detection-prediction/data). The dataset consists of the five target classes such as low/normal, medium-low, medium, medium-high, and high. The collected dataset by Rachakonda et al. (2020, 2018) consists of the following parameters such as snoring range of the user, body temperature, respiration rate, limb movement rate, eye movement, blood oxygen levels, heart rate, number of hours of sleep, and stress level. Figure 2 shows the range of the parameter values.

The used dataset is highly balanced as the dataset contains an equal number of each class. The description of the dataset variable is shown in Tables 2 and 3 showing the sample of the dataset.

**Table 2:** Dataset description.

Variable	Description	Min/Max
sr	Snoring range of a person	45.0/100
rr	Respiration rate	16.0/30.0
t	Body temperature	85.0/99.0
lm	Limb movement rate	4.0/19.0
bo	Blood oxygen levels in a person	82.0/97.0
rem	Eye movement rate	60.0/105.0
srh	Sleeping rate in hours	0.0/9.0
hr	Heart rate	50.0/85.0
sl	Stress level	0/4

**Table 3:** Sample of dataset.

sr	rr	t	lm	bo	rem	srh	hr	sl
93.8	25.68	91.84	16.6	89.84	99.6	1.84	74.2	3
91.64	25.104	91.552	15.88	89.552	98.88	1.552	72.76	3
60	20	96	10	95	85	7	60	1
85.76	23.536	90.768	13.92	88.768	96.92	0.768	68.84	3
48.12	17.248	97.872	6.496	96.248	72.48	8.248	53.12	0

**Figure 3:**
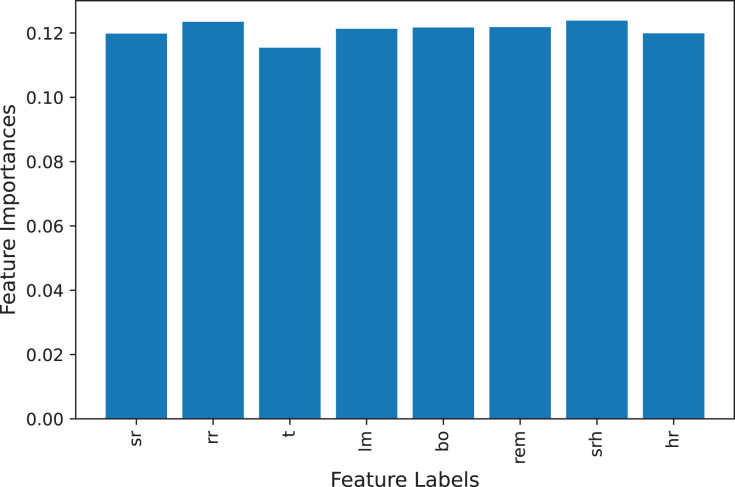
Feature importance graph.

The stress dataset consists of the eight features for the training of machine learning models as shown in Table 2. To check that, dataset features are correlated to the target class or that some features are not important to predict the stress level we deployed and calculate the feature importance. We find the feature importance using the extra tree classifier (Gupta, 2020) and the feature importance graph is shown in Fig. 3. All features present in the dataset are important for stress prediction so we used all features in the training and testing set as all features are equally important.

### Data splitting

This section contains a discussion about data splitting. We split the dataset with an 80:20 splitting ratio because the dataset size is small and we use most of the data for training purposes. While the remaining 20% of the data we used for the testing purpose. The dataset consist of a total of 630 records and 126 belong to each class. The ratio of training and testing set with respect to each target class is shown in Table 4.

**Table 4:** Target class count for training and testing sets.

Class	Training set	Testing set	Total
Low/normal (0)	99	27	126
Medium low (1)	102	24	126
Medium (2)	101	25	126
Medium high (3)	102	24	126
High (4)	100	26	126

### Machine learning models

We deployed several machine learning models in comparison with each other and selected the best in terms of performance to make a hybrid model. We used three tree-based models such as RF, GBM, and two linear models LR, and SVM. We tuned these models with their best hyper-parameters setting to achieve their best results. We tuned models between specific ranges using the grid search method and hyper-parameter setting of machine learning models is shown in Table 5.

**Table 5:** Target class count for training and testing sets.

Class	Hyper-parameters
LR	solver = liblinear, C = 2.0
SVM	kernel = ‘linear’, C = 2.0
RF	n_estimators = 200, max_depth = 50
GBM	n_estimators = 200, max_depth = 50, learning_rat = 0.2
ADA	n_estimators = 200, max_depth = 50, learning_rat = 0.2
HB	voting = soft, models = RF, GBM

### LR

LR is a statistical model used for the classification of data. LR find the relationship between dependent and independent variables (Rustam et al., 2020a). LR is used to perform computation for the probability of outcomes given an input variable. LR used the black box function Softmax to find the probabilities basis on the relationship between input variables and outcomes. Softmax function can be defined as:

(1) $$\sigma {(z)_i} = \displaystyle{{e_i^z} \over {\sum\nolimits_{u = 1}^v {e_j^z} }}$$

Here, $\sigma$ is softmax, $\vec z$ is input vector, ${e^{{z_i}}}$ input vector standard exponential function $v$ is the number of target classes, ${e^{{z_j}}}$ is output vector standard exponential function ${e^{{z_j}}}$ output vector standard exponential function.

### RF

RF is the tree-based model used for classification and regression. It is an ensemble model which combined the number of decision trees under majority voting criteria (Rustam et al., 2021). Decision trees work as weak learners in RF and then predictions from these weak learners will be combined for voting and the target class with more number predictions will be the final prediction by RF. We can define RF mathematically as:

(2) $$RF = mode\sum\limits_{n = 1}^N T re{e_i}$$

Here, *N* is the number of decision trees in RF. We used RF with 200 n_estimators in the hyper-parameters setting which means that 200 decision trees will participate in the prediction procedure.

### GBM

GBM is also an ensemble model used for regression and classification tasks (Natekin & Knoll, 2013). GBM also combined weak learners such as decision trees to make prediction processes significant. Unlike RF, GBM use boosting method, and each tree will combine sequentially. Error rate from first weak learners will be transferred to second and then further so to reduce it and get optimal training. We used GBM with 200 decision trees which means that 200 decision trees will participate in the prediction procedure and each tree is restricted to max 50 level depth using max_depth hyper-parameters which will help to reduce the complexity in learning models.

### ADA

ADA is a tree-based model similar to GBM because it also used boosting method to enhance the performance (Owusu, Zhan & Mao, 2014). We used decision trees as weak learners with ADA and trained them with 0.2 learning_rate. We used 200 decision trees under boosting methodology with 50 level depth of each tree to reduce complexity.

### SVM

A supervised machine learning algorithm that is used both for classification and regression (Rupapara et al., 2021). SVM draws a hyperplane to classify the data into their corresponding classes. It works well depending on how much data is linearly separable. It put the feature space in n dimension and then draws multiple hyperplanes with calculation and selects the best hyperplane which separates the target classes with the best margin. We used SVM with linear kernel hyper-parameter because our dataset is linearly separable.

### HB

**Figure 4:**
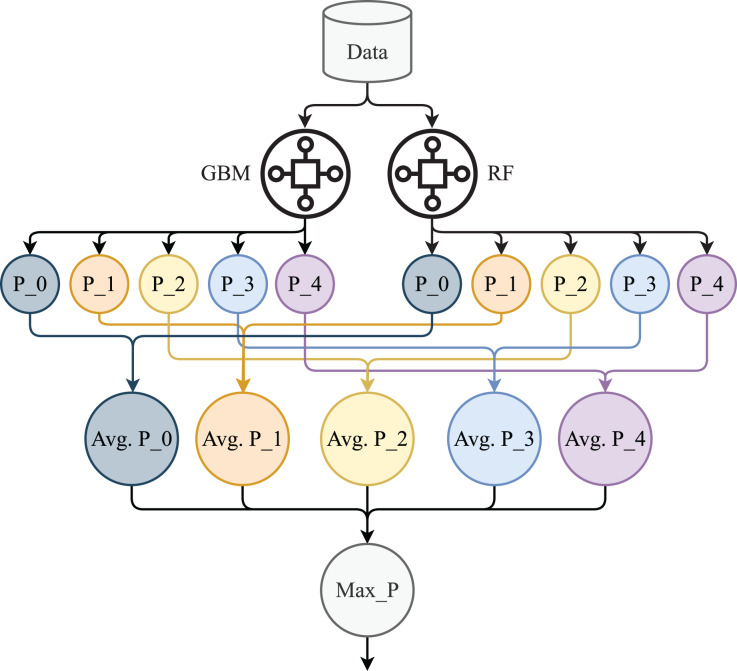
Proposed HB architecture.

The proposed HB is an ensemble model combination of GB and RF. This model we used for the prediction of stress levels. We used soft voting criteria to combine the machine learning models (Rupapara et al., 2022; Rustam et al., 2020b). The architecture of HB is shown in Fig. 4. We select GB and RF based on their performance individually in terms of accuracy and efficiency.

In Fig. 4, P_0, P_1, P_2, P_3, and P_4 are the prediction probabilities by the GBM and RF for each class. Avg P_0, Avg P_1, Avg P_2, Avg P_3, and Avg P_4 are the average probabilities for each target class while Max_p is the target class with max probability. In the proposed model, GB and RF are used with the same hyper-parameters setting such as 200 decision trees, and 50 max depth as we used in the individual model. The mathematical explanation of HB is given below:

(3) $$G\_0,G\_1,G\_2,G\_3,G\_4 = GBM(data)$$

Here, in Eq. (3), $G\_0,G\_1,G\_2,G\_3,G\_4$ are the probabilities by the GBM for 0, 1, 2, 3, 4 target classes respectively. Similarly, in Eq. (4), $R\_0,R\_1,R\_2,R\_3,R\_4$ are the probabilities by the RF for 0, 1, 2, 3, 4 target classes respectively.

(4) $$R\_0,R\_1,R\_2,R\_3,R\_4 = RF(data)$$

So, the average probabilities for each class will be:

(5) $$AvgP\_0 = \displaystyle{{G\_0 + R\_0} \over 2}$$

(6) $$AvgP\_1 = \displaystyle{{G\_1 + R\_1} \over 2}$$

(7) $$AvgP\_2 = \displaystyle{{G\_2 + R\_2} \over 2}$$

(8) $$AvgP\_3 = \displaystyle{{G\_3 + R\_3} \over 2}$$

(9) $$AvgP\_4 = \displaystyle{{G\_4 + R\_4} \over 2}$$

Now, these average probabilities will pass through the argmax function to find the target class with maximum probabilities. Here, in Eq. (17) Max_P is the target class with maximum probability which will be the final prediction.

(10) $$Max\_p = argmax\{ AvgP\_0,AvgP\_1,AvgP\_2,AvgP\_3,AvgP\_4\}$$

This computation by the model is for a single prediction. As each model is deeply involved in the prediction procedure so if one model will be a little bit wrong the second model will help to regain better results. Let’s dry run this prediction procedure on a dataset example. We take a sample from Table 3 which is 93.8, 25.68, 91.84, 16.6, 89.84, 99.6, 1.84, 74.2.

(11) $$0.1,0.4,0.5,0.8,0.5 = GBM(data)\quad and,\quad0.2,0.2,0.3,0.7,0.3 = RF(data)$$

(12) $$For\; Class\; 0 = \displaystyle{{0.1 + 0.2} \over 2} = 0.15$$

(13) $$For\; Class\; 1 = \displaystyle{{0.4 + 0.2} \over 2} = 0.3$$

(14) $$For\; Class\; 2 = \displaystyle{{0.5 + 0.3} \over 2} = 0.4$$

(15) $$For\; Class\; 3 = \displaystyle{{0.8 + 0.7} \over 2} = 0.75$$

(16) $$For\; Class\; 4 = \displaystyle{{0.5 + 0.3} \over 2} = 0.4$$

All prediction probabilities will pass through argmax function to pick the highest and according to the results, 0.75 is the highest probability belonging to class 3 so the prediction is three.

(17) $$Max\_p = argmax\{ 0.15,0.3,0.4,0.75,0.4\}$$

### Evaluation criteria

This study used four evaluation parameters to measure the performance of learning models and also used a confusion matrix for this purpose. The main evaluation parameter is accuracy which is the total number of correct predictions divided by the total number of predictions to show how much a model is correct.

(18) $$Accuracy = \displaystyle{{Total\; number\; of\ correct\; predictions} \over {Total\; number\; of\; predictions}}$$

We also used confusion matrix for the evaluation and confusion matrix consist on TP (true positive), TN (true negative), FP (false positive), and FN (false negative) (Rustam et al., 2020a). We can also find the four evaluation parameters accuracy, precision, recall, and F1 score using these confusion matrix terms such as:

(19) $$Accuracy = \displaystyle{{TP + TN} \over {TP + TN + FP + FN}}$$

(20) $$Recall = \displaystyle{{TP} \over {TP + FN}}$$

(21) $$Precision = \displaystyle{{TP} \over {TP + FP}}$$

(22) $$F1Score = \displaystyle{{Precision*\,Recall} \over {Precision + Recall}}$$

## Results

This section contains the results of machine learning and deep learning models for stress detection. All experiments have been done on Core i7 7th generation machine with windows operating system. We used Jupyter Notebook with Python language to implement machine learning models. All models are evaluated in terms of accuracy, precision, recall, and F1 Score.

### Results of machine learning models

This section contains the results of machine leanings models such as RF, LR, SVM, GBM, ADA, and proposed HM. The performance of all learning models is good just the ADA is low in accuracy score. Tree-based ensemble models RF and GB outperform with a significant accuracy score of 0.99 accuracies while linear models SVM and LR both achieved 0.95 accuracy scores respectively. The performance of LR and SVM is not good because linear models required a large dataset with a large feature set. ADA is not good in terms of accuracy score and other evaluation parameters because it’s a boosting algorithm that combines the decision trees sequentially so training of the model required a large dataset.

**Figure 5:**
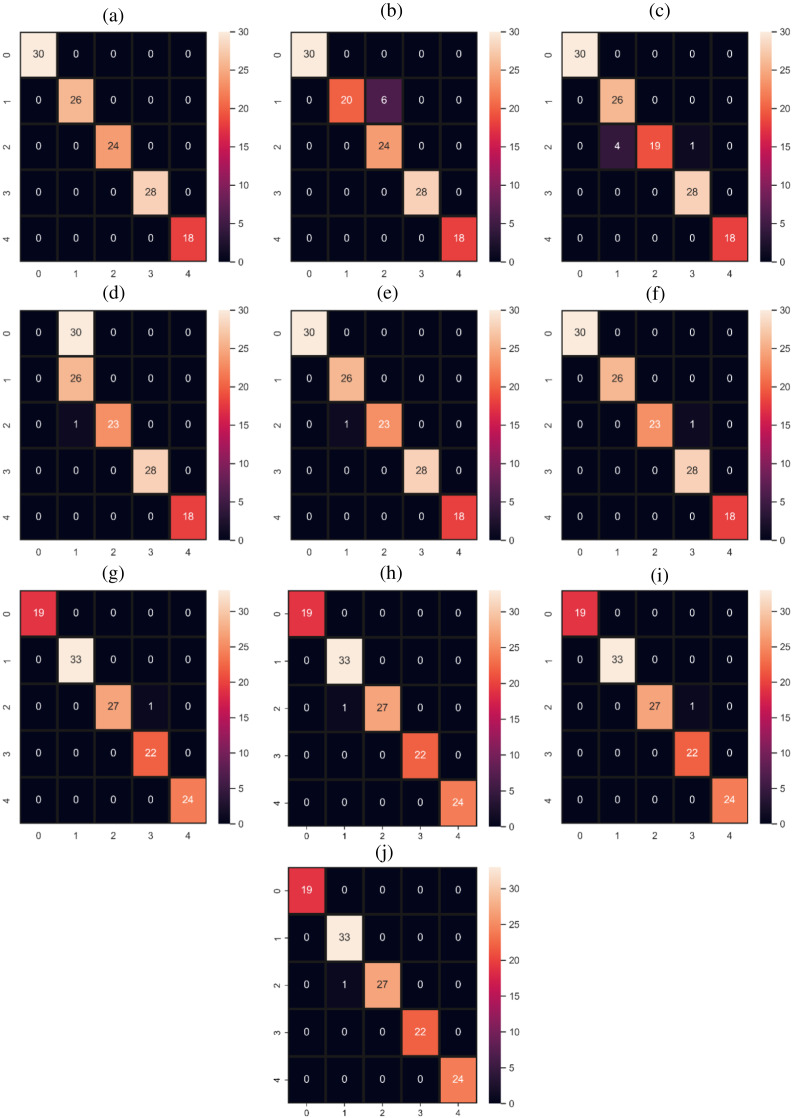
Confusion matrices for machine learning and deep learning models.

Tables 6–11 showing the results of all models. The proposed model HB outperforms all individual models with a 100% accuracy score. The proposed model HB is significant in comparison to all other used models because we combine the best performer RF and GBM under soft voting criteria. Both individually perform well and give only one wrong prediction by each as shown in Fig. 5. One model weakness is another model strength as RF predicts the medium stress (2) as medium-high stress (3) while the GBM model GBM model predicts medium stress (2) as medium-low stress (1). When we combined both models they make better calculations and resolve the issue of one wrong prediction with 100% correct predictions. The performance of the ADA model is poor for the 0 target class as shown in Table 8. It achieved 0.00 scores in terms of all evaluation parameters for 0 target class. ADA model required some large dataset to get a good fit our used dataset is small in size so its overall performance is poor as well as for 0 target class.

**Table 6:** Results obtained using RF.

Accuracy	Class	Precision	Recall	F1 score
0.99	0	1.00	1.00	1.00
	1	1.00	1.00	1.00
	2	1.00	0.96	0.98
	3	0.97	1.00	0.98
	4	1.00	1.00	1.00
	Macro avg	0.99	0.99	0.99
	Weighted avg	0.99	0.99	0.99

**Table 7:** Results obtained using GBM.

Accuracy	Class	Precision	Recall	F1 score
0.99	0	1.00	1.00	1.00
	1	1.00	0.96	0.98
	2	0.97	1.00	0.98
	3	1.00	1.00	1.00
	4	1.00	1.00	1.00
	Macro avg	0.99	0.99	0.99
	Weighted avg	0.99	0.99	0.99

**Table 8:** Results obtained using ADA.

Accuracy	Class	Precision	Recall	F1 score
0.83	0	0.00	0.00	0.00
	1	0.50	1.00	0.67
	2	0.97	1.00	0.99
	3	1.00	0.96	0.98
	4	1.00	1.00	1.00
	Macro avg	0.69	0.79	0.73
	Weighted avg	0.74	0.83	0.77

**Table 9:** Results obtained using LR.

Accuracy	Class	Precision	Recall	F1 score
0.96	0	1.00	1.00	1.00
	1	0.87	1.00	0.93
	2	1.00	0.79	0.88
	3	0.97	1.00	0.98
	4	1.00	1.00	1.00
	Macro avg	0.97	0.96	0.96
	Weighted avg	0.96	0.96	0.96

**Table 10:** Results obtained using SVM.

Accuracy	Class	Precision	Recall	F1 score
0.95	0	1.00	1.00	1.00
	1	1.00	0.77	0.87
	2	0.80	1.00	0.89
	3	1.00	1.00	1.00
	4	1.00	1.00	1.00
	Macro avg	0.96	0.95	0.95
	Weighted avg	0.96	0.95	0.95

**Table 11:** Results obtained using HB model.

Accuracy	Class	Precision	Recall	F1 score
1.00	0	1.00	1.00	1.00
	1	1.00	1.00	1.00
	2	1.00	1.00	1.00
	3	1.00	1.00	1.00
	4	1.00	1.00	1.00
	Macro avg	1.00	1.00	1.00
	Weighted avg	1.00	1.00	1.00

**Figure 6:**
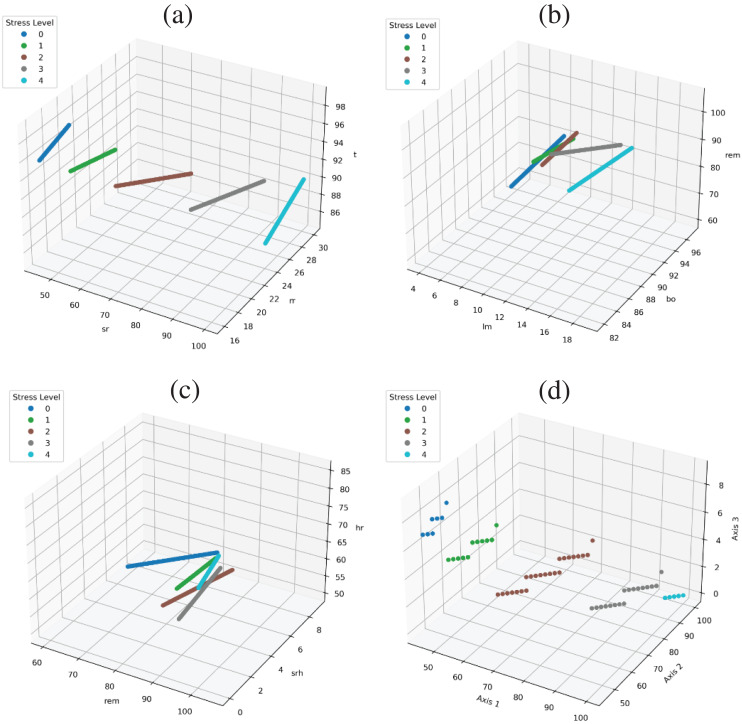
Feature space to show the correlation in target class and data variables.

The performance of models is too significant because of the dataset feature correlation with the target class. As shown in Fig. 6, all targets are linearly separable. Figure 6A show feature space using the ‘sr’, ‘rr’, ‘t’ and all target are clearly separable. Figure 6B shows the feature space using the ‘lm’, ‘bo’, ‘rem’ variables are there is a little bit of overlap in the target class with these three variables, and in Fig. 6C we used ‘rem’, ‘srh’, ‘hr’ variables and there is still little overlapping but when we used all feature together than all target are linearly separable and there is a good correlation between features and target classes as shown in Fig. 6D.

### Deep learning results

This section contains the results of deep learning models in comparison with used machine learning models. These deep learning models are used with their state-of-the-art architecture taken from previous studies worked on similar kinds of datasets (Rupapara et al., 2022). The architecture of the used models is shown in Table 12. All models start with their embedding layer consisting of vocabulary size 1,000 and output size 100. Each model consists of a dropout layer with a dropout size of 0.5 which helps to reduce complexity in learning models (Reshi et al., 2021). In the end, each model compiles with categorical cross-entropy loss function because of multi-class data and adam optimizer. Models are fitted with a batch size of 8 and 100 epochs.

**Table 12:** The architecture of used deep learning models.

RNN	LSTM
Embedding(1,000,100)	Embedding(1,000,100)
Dropout(0.5)	Dropout(0.5)
SimpleRNN(64)	LSTM(16)
Dense(32)	Dense(32)
Dense(5, activation=‘softmax’)	Dense(5, activation=‘softmax’)
CNN	CNN-LSTM
Embedding(1,000,100)	Embedding(5,000,100)
Dropout(0.5)	Dropout(0.5)
Conv1D(64, 2, activation=‘relu’)	Conv1D(64, 2, activation=‘relu’)
MaxPooling1D(pool size=2)	MaxPooling1D(pool size=2)
Flatten()	LSTM(32)
Dense(32)	Dense(16)
Dense(5, activation=softmax)	Dense(5, activation=softmax)
loss=‘categorical crossentropy’,	optimizer=‘adam’, epochs100

**Figure 7:**
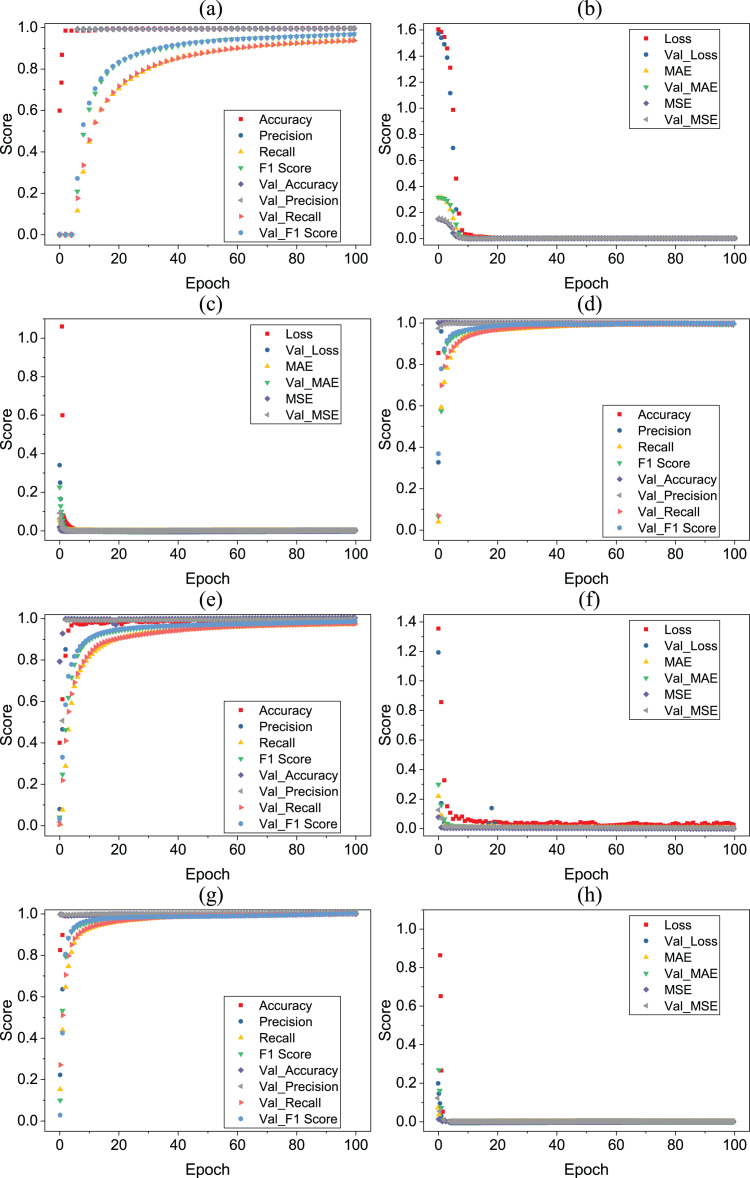
(A–H) Deep learning models per epochs scores.

Tables 13–16 showing the results of deep learning models. According to the results, all deep learning models equally perform well with a 0.99 accuracy score. All deep learning models give wrong predictions for medium stress (2) and medium-low stress (1) target classes. Figure 7 shows the accuracy loss graph for each deep learning model according to per epochs.

**Table 13:** Results obtained using LSTM model.

Accuracy	Class	Precision	Recall	F1 score
0.99	0	1.00	1.00	1.00
	1	1.00	1.00	1.00
	2	1.00	0.96	0.98
	3	0.96	1.00	0.98
	4	1.00	1.00	1.00
	Macro avg	0.99	0.99	0.99
	Weighted avg	0.99	0.99	0.99

**Table 14:** Results obtained using CNN model.

Accuracy	Class	Precision	Recall	F1 score
0.99	0	1.00	1.00	1.00
	1	0.97	1.00	0.99
	2	1.00	0.96	0.98
	3	1.00	1.00	1.00
	4	1.00	1.00	1.00
	Macro avg	0.99	0.99	0.99
	Weighted avg	0.99	0.99	0.99

**Table 15:** Results obtained using RNN model.

Accuracy	Class	Precision	Recall	F1 score
0.99	0	1.00	1.00	1.00
	1	0.97	1.00	0.99
	2	1.00	0.96	0.98
	3	1.00	1.00	1.00
	4	1.00	1.00	1.00
	Macro avg	0.99	0.99	0.99
	Weighted avg	0.99	0.99	0.99

**Table 16:** Results obtained using CNN-LSTM model.

Accuracy	Class	Precision	Recall	F1 score
0.99	0	1.00	1.00	1.00
	1	1.00	1.00	1.00
	2	1.00	0.96	0.98
	3	0.96	1.00	0.98
	4	1.00	1.00	1.00
	Macro avg	0.99	0.99	0.99
	Weighted avg	0.99	0.99	0.99

Deep learning models are efficient as well as individual machine learning models even on small datasets also but they are not efficient in terms of computational time. Deep learning models take more time for computation while machine learning models take low time. Our proposed models outperform deep learning both in terms of accuracy and efficiency with low computational time and high accuracy scores as shown in Table 17.

**Table 17:** Computational cost of learning models.

Model	Time	Model	Time
RF	0.281	LSTM	68.896
LR	0.111	RNN	133.72
GBM	0.962	CNN	91.002
ADA	5.998	CNN-LSTM	124.363
SVM	0.076	HB	1.31

### K-fold cross-validation results

To show the significance of our proposed approach we have also done k-fold cross-validation using all models on the used dataset. We used 10 fold in k-fold validation and the results of 10 fold cross validation are shown in Table 18. Performance of all models is approximately similar as was in train test split testing validation but Adaboost is poorer in 10 Fold cross-validation with 0.67 mean accuracy and 0.12 standard deviation (SD). Our proposed models outperform other models in 10 fold cross validation approach also with 1.00 mean accuracy and 0.00 SD which show the significance of the proposed approach.

**Table 18:** 10-fold cross validation results.

Model	Accuracy	SD	Model	Accuracy	SD
RF	0.99	+/−0.01	LSTM	0.99	+/−0.01
LR	0.95	+/−0.03	RNN	0.99	+/−0.03
GBM	0.99	+/−0.02	CNN	0.99	+/−0.02
ADA	0.67	+/−0.12	CNN-LSTM	0.99	+/−0.01
SVM	0.96	+/−0.04	HB	1.00	+/−0.00

### Comparison with other studies

In this section, we compared our proposed approach with previous studies approaches. We select studies from the literature that have done work on stress detection and worked on hybrid models. We deployed their approaches on our used dataset to make a fair comparison. We implement previous studies’ approaches in the same environment as we deployed our approach. Then we perform the experiment on the same dataset which we used in this study for our proposed approach validation. We used study Salazar-Ramirez et al. (2018) for comparison because it also has worked on stress detection and used Gaussian SVM as a learning model. Similarly, Alberdi et al. (2018) and Lim et al. (2018) also have done work on stress detection and deployed SVM and ANN as learning models respectively. Rupapara et al. (2022) used LVTrees for blood cancer prediction. They combined LR, SVM, ETC for better results and we deploy this model on our used dataset. The comparison with other studies methods shown in Table 19.

**Table 19:** Comparison with other studies.

Ref.	Model	Accuracy	Precision	Recall	F1 score
Salazar-Ramirez et al. (2018)	Gaussian SVM	0.94	0.94	0.94	0.94
Alberdi et al. (2018)	SVM	0.95	0.94	0.95	0.94
Lim et al. (2018)	ANN	0.98	0.98	0.98	0.98
Rupapara et al. (2022)	LVTrees	0.97	0.97	0.97	0.97
This study	HB	1.00	1.00	1.00	1.00

### Proposed approach results using another dataset

**Figure 8:**
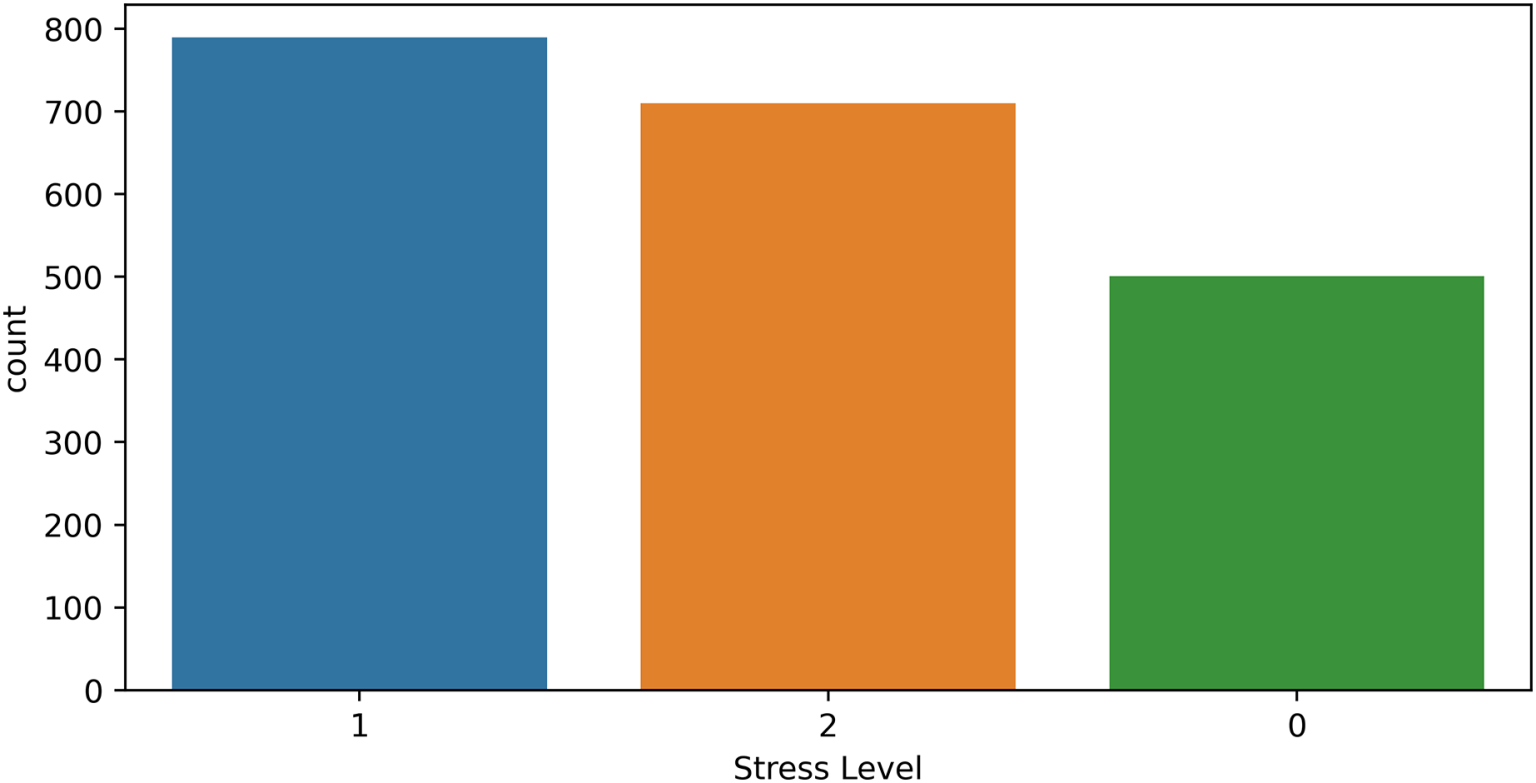
Human stress detection dataset target ratio.

We experimented with another dataset to show the significance of the proposed approach. We used the “Human Stress Detection” dataset available on the public data repository, Kaggle (Rachakonda, 2022). This dataset consists of physiological features such as “Humidity, Temperature, Step count” and three target classes low stress (0), normal stress (1), and high stress (2). The dataset contains the total 2,001 samples and the ratio for each target class in the dataset is shown in Fig. 8.

We performed experiments using all models and the results are shown in Table 20. Results show that tree-based models are significant on this dataset with 100% accuracy and the HB model also outperforms in terms of all evaluation parameters because it’s a combination of tree-based models. Linear models such as LR and SVM are also good with 0.95 and 0.98 accuracy scores. All deep learning models are good with a 0.99 score in terms of all evaluation parameters. Our all proposed models are significant with the highest accuracy on both used datasets in this study.

**Table 20:** Model performance comparison on the “human stress detection” dataset.

Model	Accuracy	Precision	Recall	F1 score
RF	1.00	1.00	1.00	1.00
LR	0.95	0.95	0.95	0.95
GBM	1.00	1.00	1.00	1.00
ADA	1.00	1.00	1.00	1.00
SVM	0.98	0.98	0.98	0.98
LSTM	0.99	0.99	0.99	0.99
CNN	0.99	0.99	0.99	0.99
RNN	0.99	0.99	0.99	0.99
CNN-LSTM	0.99	0.99	0.99	0.99
HB	1.00	1.00	1.00	1.00

### Statistical T-test

We have done a statistical T-test to show the significance of the proposed approach. T-test rejects or accepts the null hypothesis based on the given two inputs. We compared the results of the proposed HB with all other used methods and find the T-score and critical value (CV). If the T score is less or equal to CV then its T-test accepts the null hypothesis else rejects the null hypothesis and accepts the alternative hypothesis.

- Accepted null hypothesis means there is no statistical difference in compared results.
- Accepted alternative hypothesis means there is a statistical difference in compared results.

Table 21 show the T-test value for each compared approach. In each case, T-test reject the null hypothesis and accepted the alternative hypothesis which means that HB is statistically significant as compared to other techniques. T-test gives 7.20 ${e^{ - 17}}$ CV and 2.3 T-score in each case which means a T-score greater than a CV in each compared case.

**Table 21:** Statistical T-test results.

Case	T-score	CV	Hypothesis	Case	T-score	CV	Hypothesis
HB *vs*. RF	2.3	7.20 ${e^{ - 17}}$	Reject	HB *vs*. LSTM	0.99	7.20 ${e^{ - 17}}$	Reject
HB *vs*. LR	9.2	7.20 ${e^{ - 17}}$	Reject	HB *vs*. RNN	2.3	7.20 ${e^{ - 17}}$	Reject
HB *vs*. GBM	2.3	7.20 ${e^{ - 17}}$	Reject	HB *vs*. CNN	2.3	7.20 ${e^{ - 17}}$	Reject
HB *vs*. ADA	6.2	7.20 ${e^{ - 17}}$	Reject	HB *vs*. CNN-LSTM	2.3	7.20 ${e^{ - 17}}$	Reject
HB *vs*. SVM	9.2	7.20 ${e^{ - 17}}$	Reject				

## CONCLUSION

This study proposed an approach for stress detection using machine learning and deep learning techniques. A hybrid approach was constructed to achieve high accuracy and efficiency. We proposed a hybrid model HB which is the combination of two tree-based ensemble models RF and GBM. Both models can perform well on small datasets because of their ensemble architecture. In combination they further improve the performance and achieved the 100% accuracy score. This significant performance is because of the soft voting criteria in the HB model which find the probability for each class using all combined models and the class with the highest probability is the final prediction by HB. We concluded that for the small dataset tree-based models are more accurate as compared to linear models because they required a large dataset for a good fit. We also concluded that the ensemble of models can perform better as compared to an individual model because it gives more deep computation for the prediction. Deep learning models can be good for large datasets but in terms of computational cost, they can be costly as compared to machine learning models. There is another conclusion of the study which is that the machine learning models can give the highest accuracy when the dataset features are correlated to the target classes as we used in this study. In future work, we will work on dataset collection as the used dataset is very small in size so our future directions are towards the collection of more data.

## Supplemental Information

10.7717/peerj-cs.1154/supp-1 Supplemental Information 1 Data files for stress detection.

10.7717/peerj-cs.1154/supp-2 Supplemental Information 2 Code experiments.

## Additional Information and Declarations

### Data Availability

The following information was supplied regarding data availability: The code is available in the Supplemental File. The data from Kaggle (https://www.kaggle.com/datasets/laavanya/human-stress-detection-in-and-through-sleep, owned by Laavanya Rachakonda from the University of North Carolina Wilmington) is available in the Supplemental File.
